# A high-resolution tomography (HRCT) scan showing classical “air crescent sign” in a case of pulmonary aspergilloma

**DOI:** 10.11604/pamj.2023.45.15.39021

**Published:** 2023-05-04

**Authors:** Puja Upadhyay, Ulhas Jadhav

**Affiliations:** 1 Department of Respiratory Medicine, Jawahar Lal Nehru Medical College, Datta Meghe Institute of Higher Education and Research, Sawangi (Meghe), Wardha, Maharashtra, India

**Keywords:** Aspergilloma, air crescent sign, monod sign

## Image in medicine

A 45-year-old male presented to the hospital with complaints of hemoptysis for the last 3 weeks. He also had a cough with mucoid expectoration for 3 weeks. He denied a history of fever/loss of weight and appetite. The patient had a past history of sputum-positive pulmonary tuberculosis 20 years back for which he had taken AKT for 6 months. A suspicion of reactivation of pulmonary tuberculosis was made and sputum was sent for AFB (acid fast bacilli) and Nucleic Acid Amplification Test (NAAT) which was negative. On further investigation high-resolution computed tomography (HRCT) was done which revealed an irregularly shaped large cavitatory lesion

in the left upper lobe. The cavity was filled with large soft tissue surrounded by air crescent suggestive of fungal ball formation (aspergilloma). This HRCT revealed a classic “air crescent sign” (marked by an arrow) seen in aspergilloma but is also found in pulmonary tuberculosis, hydatid cyst, and pulmonary abscess. In aspergilloma, this mass usually moves within the cavity when the patient changes position and the sign is called “Monod sign”. Aspergilloma most commonly occurs in the cavity formed secondary to tuberculosis and is a common sequelae of pulmonary tuberculosis. Most aspergilloma are asymptomatic and hemoptysis secondary to reactive vascular granulation tissue is the most common presentation. For this patient bronchial artery embolization (BAE) was done and he was started on antifungal treatment. Though in patients with recurrent or massive hemoptysis, surgical resection remains the gold standard. In our case, the patient did not have any more episodes of hemoptysis after BAE and was discharged in stable condition with the advice of regular follow-up.

**Figure 1:**
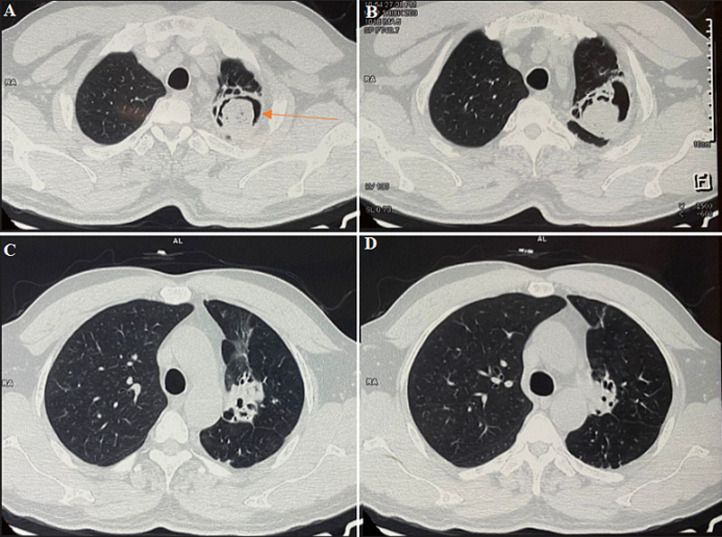
A,B,C,D) high resolution computed tomography (HRCT) of the thorax showing air crescent sign

